# Shared genetic basis and spatial cellular atlas of psoriasis and metabolic syndrome

**DOI:** 10.1371/journal.pone.0358472

**Published:** 2026-09-15

**Authors:** Guo Liu, Fengjuan Gong, Guanhu Yang, Yu Li, Yanjiao Ji, Xinyue Chen, Chao Wang, Qinghua Luo

**Affiliations:** 1 Qionglai Hospital of Traditional Chinese Medicine, Chengdu, China; 2 Faculty of Chinese Medicine, Macau University of Science and Technology, MacauChina; 3 Sichuan Academy of Chinese Medicine Sciences, Chengdu, China; 4 Clinical Medical College, Jiangxi University of Chinese Medicine, Nanchang, China; MAIWP International University, MALAYSIA

## Abstract

**Background:**

Psoriasis (PS) and metabolic syndrome (MetS) frequently co-occur. Characterizing their shared genetic architecture and spatially enriched cellular populations may clarify the context of their co-occurrence and generate hypotheses for functional validation.

**Methods:**

We integrated genome-wide association study (GWAS) summary statistics for PS, MetS, and five related components with spatially resolved single-cell transcriptomic data. Global and local genetic correlations were assessed using linkage disequilibrium score regression, genetic covariance analysis, high-definition likelihood, and local analysis of variant association. A bivariate causal mixture model quantified polygenic overlap. Conditional/conjunctional false discovery rate and composite-null pleiotropy analyses identified shared susceptibility loci. Finally, gsMap evaluated trait-associated enrichment across annotated embryonic tissues at single-cell resolution.

**Results:**

Genetic approaches identified significant genome-wide correlations and polygenic sharing between PS, MetS, and its components. Local and cross-trait analyses identified region-specific signals and cross-validated shared loci. gsMap revealed trait-specific tissue enrichment. PS showed the strongest enrichment in the epidermis (pCauchy = 1.0573 × 10  ^−^ ⁴), adipose tissue (pCauchy = 1.5366 × 10 ^−^ ⁴), and liver (pCauchy = 1.0167 × 10 ^−^ ³). Across MetS, FBG, HDL-C, hypertension, and TG, enriched regions mainly involved the liver, adipose tissue, and epidermis. WC enrichment was predominantly observed in adipose tissue (pCauchy = 1.7823 × 10 ^−^ ⁴), with no significant liver or epidermal enrichment.

**Conclusion:**

Integrating GWAS with single-cell transcriptomic and spatial information characterized shared genetic architecture between PS and MetS-related phenotypes and their spatial enrichment patterns. These findings provide a framework for generating testable hypotheses about comorbidity biology and guiding future functional and clinical validation.

## 1. Introduction

Psoriasis (PS) refers to a chronic, relapsing inflammatory skin disorder driven by immune dysregulation, characterized by sharply demarcated erythematous plaques covered with silvery-white scales [1]. PS is among the most prevalent immune-mediated conditions worldwide, affecting approximately 2–3% of the global population, with an estimated 125 million individuals currently diagnosed [1]. The clinical presentation of PS is heterogeneous, with plaque PS (chronic plaque type) representing the predominant subtype, accounting for 80–90% of all cases. It typically manifests as well-circumscribed erythematous plaques coated with silvery-white scales and is frequently accompanied by pruritus [2]. Lesions most commonly arise on the scalp, extensor surfaces (elbows and knees), lumbosacral region, and nails, and a subset of patients exhibits joint involvement [3]. Evidence indicates that approximately 30% of individuals with PS develop psoriatic arthritis, resulting in joint pain, stiffness, and impaired functional capacity [4]. Moreover, PS is frequently associated with multiple comorbidities, among which metabolic syndrome (MetS) requires particular attention, with reported prevalence ranging from 14.3% to 50% [5]. MetS denotes a constellation of metabolic disturbances, including dysglycemia, hyperlipidemia, central obesity, and hypertension [6]. The genetic correlation between PS and MetS has garnered increasing interest in recent years [5,7]. Recent genetic studies published in 2026 have further extended this evidence. A cross-trait genome-wide association study (GWAS) identified shared loci and pleiotropic genes connecting PS with MetS and several of its component traits [8]. The associated genes were enriched in immune–inflammatory, transcriptional, autophagic, and lipid–cholesterol pathways [8]. Complementary analyses linked psoriasis susceptibility to adverse cardiovascular–kidney–metabolic status and genetically predicted unsaturated fatty-acid profiles [9,10]. Together, these findings support shared immunometabolic susceptibility while highlighting heterogeneity across individual metabolic components. Traditional epidemiological investigations, when examining the mechanisms underlying disease coexistence, are often restricted in their causal interpretability due to confounding, sampling bias, and limited follow-up [11]. GWAS complement epidemiological evidence by identifying inherited genetic associations at scale; however, they rely on assumptions concerning phenotype definition, population structure, linkage disequilibrium, and dataset comparability and do not by themselves establish causal mechanisms [12].

Technological advances in functional genomics have enabled integrative analytical strategies to investigate how genetic variants influence cellular processes. Because no single method captures all dimensions of cross-trait genetic sharing, we used a complementary, multi-level analytical framework. LDSC [13], GNOVA [14], and HDL [15] were jointly applied to estimate genome-wide genetic correlations under different statistical frameworks, allowing the direction and magnitude of the global estimates to be assessed for robustness. Because global estimates may obscure region-specific signals with opposing directions, LAVA was used to identify local genetic correlations and characterize regional heterogeneity [16]. MiXeR complemented correlation-based analyses by quantifying shared and trait-specific polygenic components, including overlap that may persist despite modest genome-wide genetic correlation [17]. At the locus level, condFDR leverages cross-trait enrichment to improve discovery, whereas conjFDR prioritizes variants jointly associated with both traits [18]. PLACO provides a complementary pleiotropy test under the composite null hypothesis, reducing dependence on a single locus-discovery assumption [19]. Finally, gsMap integrates GWAS evidence with spatial omics data to localize trait-associated signals to specific cell types and spatial niches [20]. Together, these complementary methods connect genome-wide sharing, regional heterogeneity, polygenic overlap, pleiotropic loci, and spatial cellular localization.

Guided by this rationale, this study applied the multi-level framework to characterize genome-wide and regional genetic sharing between PS and MetS. We further quantified polygenic overlap, prioritized pleiotropic candidate genes, and mapped trait-associated signals to spatially resolved cellular contexts. This integration provided a coherent evidence chain from genetic association to functional and spatial interpretation, generating testable hypotheses about PS–MetS comorbidity and priorities for future functional validation.

## 2. Materials and methods

### 2.1. GWAS Data

PS was defined in FinnGen release R12 (endpoint: L12_PSORIASIS) as a binary registry-based endpoint using ICD-10 codes L40.0–L40.9 [21]. Participants were genotyped using Illumina and Affymetrix arrays and imputed using the Finnish population-specific SISu reference panel. The MetS GWAS (ebi-a-GCST009602) applied Lind’s harmonized NCEP ATP III criteria [22], requiring at least three of five components: blood pressure ≥130/85 mmHg or antihypertensive treatment; serum glucose ≥6.1 mmol/L or antidiabetic treatment; TG ≥ 1.7 mmol/L; WC > 102 cm in men or >88 cm in women; and HDL-C < 1.0 mmol/L in men or <1.3 mmol/L in women. Although this definition shares the five-component structure of the 2009 IDF/AHA–NHLBI framework [23], it uses different WC and glucose thresholds. Thus, binary MetS refers specifically to the Lind UK Biobank phenotype. FBG, HDL-C, TG, and WC were analyzed as continuous traits, whereas hypertension was analyzed as a binary trait. Further details on dataset sources, sample sizes, trait definitions, summary-statistics versions and formats, and ancestry are provided in Table 1 in S1 Table.

**Fig 1:**
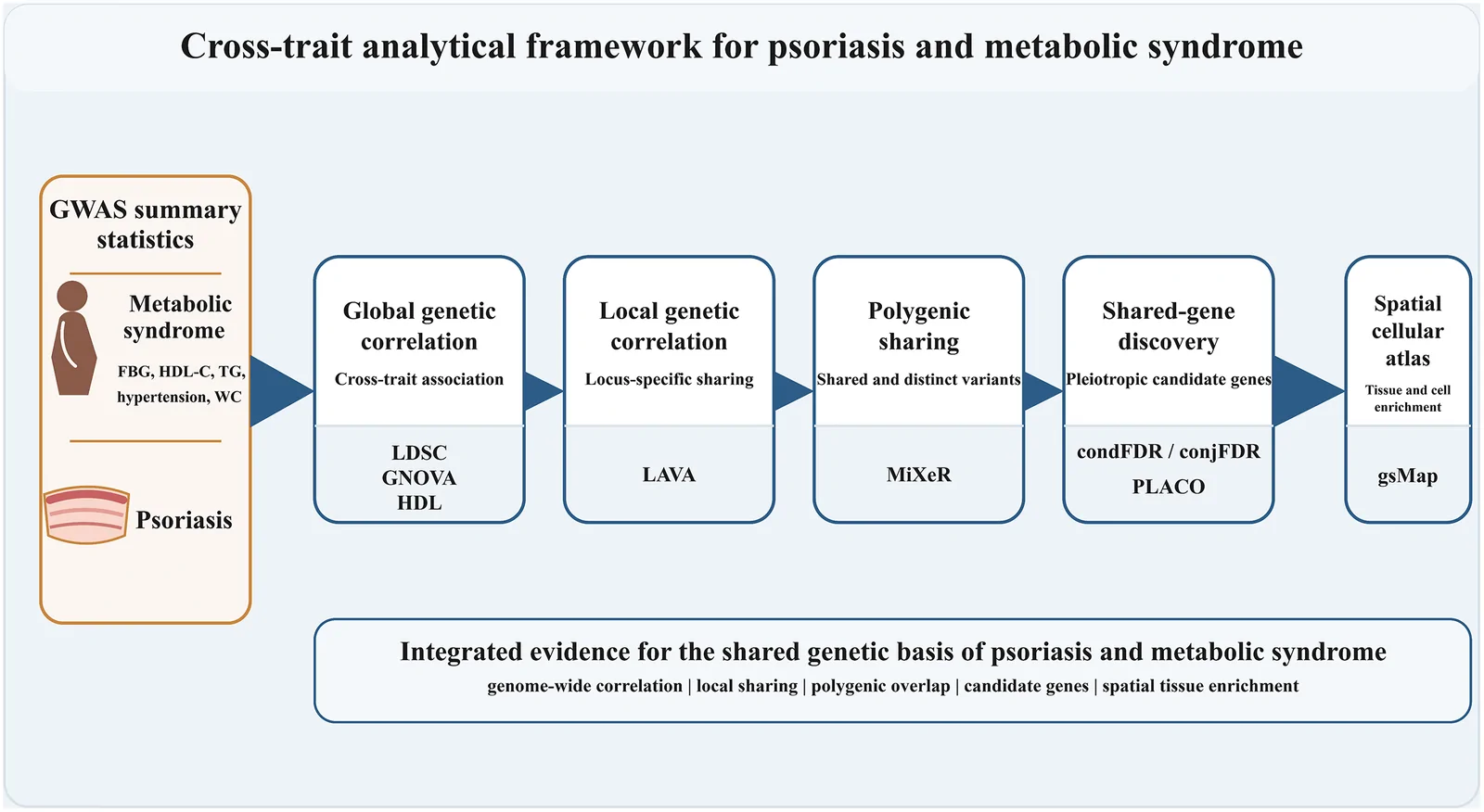
Visual overview of the experimental design. The graphic was constructed with BioRender. LDSC, linkage disequilibrium score regression; GNOVA, genetic covariance analysis; HDL, high-definition likelihood; LAVA, local analysis of variant association; MiXeR, bivariate causal mixture model; condFDR/conjFDR, conditional/conjunctional false discovery rate; gsMap, genetically informed spatial mapping of cells for complex traits.

All GWAS summary statistics used in this study originated from previously published datasets that had obtained institutional ethical approval, participant consent, and underwent strict quality control. All source GWASs applied sample- and variant-level quality control and cohort-specific genotype imputation. FinnGen used the SISu reference panel, whereas UK Biobank primarily used the HRC and UK10K/1000 Genomes reference panels. In this study, summary statistics were harmonized to hg19/GRCh37, and single-nucleotide polymorphisms (SNPs) with missing or duplicate rsIDs were removed. No additional minor allele frequency (MAF) or P-value filter was applied during data import. Variants within the extended major histocompatibility complex (MHC) region on chromosome 6 (chr6:25,000,000–34,000,000, hg19/GRCh37) were excluded during preprocessing before all downstream analyses; for LAVA, LD blocks overlapping this interval were removed. This conservative preprocessing step was applied to prevent strong psoriasis-associated HLA signals and complex long-range LD from dominating estimates of the shared genetic architecture. Analyses were restricted to individuals of European ancestry. Exact cohort overlap was unavailable, although overlap among UK Biobank-derived datasets was possible. The complete analytical workflow is shown in Fig 1. Software versions, repository commits, reference panels, key parameters, genomic exclusion regions, and non-default settings for all analytical tools are summarized in Table 2 in S1 Table.

### 2.2. Global genetic correlation analyses

Three complementary approaches, LDSC (https://github.com/bulik/ldsc), GNOVA (https://github.com/xtonyjiang/GNOVA), and HDL (https://github.com/zhenin/HDL), were used to estimate genome-wide genetic correlations between PS and MetS from GWAS summary statistics. LDSC estimates standardized genetic covariance by relating SNP association statistics to LD scores without requiring individual-level data and is robust to sample overlap [13]. GNOVA uses weighted genome-wide covariance estimation with explicit correction for sample overlap, improving precision in datasets with overlapping samples or heterogeneous structures [14]. HDL applies full-likelihood optimization to genome-wide LD matrices with eigenvalue-based dimension reduction, reducing standard errors by approximately 60% relative to LDSC [15]. Agreement across the three approaches was used to assess the robustness of the genetic-correlation estimates. All analyses used summary statistics after excluding the extended MHC region (chr6:25,000,000–34,000,000, hg19/GRCh37). The reported estimates therefore represent genome-wide genetic correlations outside this predefined interval.

### 2.3. Local genetic correlation analyses

The LAVA (https://github.com/josefin-werme/LAVA) method initially partitions the entire genome into multiple independent LD blocks according to LD structural characteristics, ensuring that genetic variants within each block remain highly correlated while maintaining genetic independence across blocks [16]. On this basis, GWAS summary statistics for PS and MetS, together with LD information matrices derived from European ancestry reference populations, are incorporated to model and estimate the local genetic variance–covariance structure for both phenotypes within each predefined genomic region. Local genetic correlation coefficients are subsequently calculated to quantitatively delineate the extent to which specific genomic regions contribute to disease comorbidity. Through statistical testing, LAVA enables the identification of genomic regions showing significant local genetic correlations, thereby characterizing the regional distribution and heterogeneity of genetic overlap between PS and MetS. In comparison with global genetic correlation analyses, the principal advantage of LAVA lies in its capacity to detect region-specific correlation patterns that may be obscured by genome-wide estimates and to prioritize associated genomic regions for subsequent fine-mapping and functional investigation.

### 2.4. MiXeR

MiXeR (https://github.com/precimed/mixer) applies a bivariate causal mixture modeling framework to systematically quantify the extent of polygenic overlap between PS and MetS using GWAS summary statistics [17]. By fitting the distribution of GWAS effect sizes, this method partitions genome-wide genetic variation into three independent components: PS-specific causal variants, MetS-specific causal variants, and variants shared by both conditions. The quantitative parameters for each component are inferred through maximum likelihood estimation, and the Dice overlap coefficient is subsequently computed to measure genetic similarity (ranging from 0 to 1, indicating complete independence to complete overlap, respectively). This framework facilitates precise quantification of shared and disease-specific genetic architecture at the SNP level, thereby providing quantitative evidence for identifying pleiotropic candidate genes contributing to comorbidity.

### 2.5. condFDR/conjFDR Analysis

The condFDR/conjFDR (https://github.com/precimed/pleiofdr) framework was implemented as originally described by Andreassen et al. [24] and subsequently extended and reviewed by Smeland et al. [18]. This approach incorporates genetic information from a secondary trait to increase the statistical power of the primary trait, thereby detecting association signals that may be missed in conventional GWAS analyses. The analytical procedure proceeded as follows: GWAS summary statistics from both diseases were initially integrated, followed by quality control and LD correction. Conditional quantile–quantile plots were then generated to evaluate the enrichment of genetic signals for the primary trait (e.g., PS) conditional on the secondary trait (e.g., MetS), where stronger enrichment was reflected by greater leftward deviation of the curve. Based on the enrichment distribution, the condFDR for each SNP was computed using the empirical cumulative distribution function, with correction for genomic inflation applied. Statistical independence was ensured by randomly selecting representative SNPs from LD blocks. The analysis was subsequently repeated with the roles of primary and secondary traits reversed, and the larger of the bidirectionally obtained condFDR values was defined as the conjFDR. Shared loci were identified using a conjFDR threshold of < 0.05. Through its conditional analytical structure, this method increases detection power by integrating information from correlated traits and enables identification of pleiotropic genetic signals without requiring consistency in effect direction [18].

### 2.6. PLACO

To systematically identify shared genetic loci between PS and MetS, PLACO (https://github.com/RayDebashree/PLACO) [19] was applied in this study. Unlike conventional approaches, PLACO is anchored in a composite null hypothesis testing framework, in which the null holds when a genetic variant exerts no effect on either trait or influences only one; otherwise, the variant is classified as pleiotropic. In implementation, PLACO extracts effect estimates and standard errors for each SNP from the GWAS summary statistics of both diseases and constructs a joint test statistic based on standardized effect values (Z-scores). This statistic incorporates covariance information between the traits to correct for bias introduced by sample overlap. A central methodological feature of PLACO is its avoidance of any requirement for prespecified effect direction consistency; instead, significant loci are detected at a threshold of *P* < 5 × 10 ^−^ ⁸ by optimizing across all possible combinations of effect directions. This framework enables simultaneous detection of both concordant and discordant pleiotropy, thereby increasing the sensitivity of cross-trait association identification. Moreover, this method relies solely on GWAS summary statistics and maintains robust performance under conditions of sample overlap, effect-size heterogeneity, and varying genetic correlation [19].

The SNP2GENE functional module of the FUMA [25] platform was used to conduct detailed gene annotation for genetic variants identified through the condFDR/conjFDR and PLACO analyses.

### 2.7. Candidate-gene annotation and prioritization

Variants meeting the conjFDR (< 0.05) or PLACO (*P* < 5 × 10 ^−^ ⁸) threshold were mapped to genes using the SNP2GENE module of FUMA [25]. For focused biological interpretation, candidate genes were prioritized using a hierarchical decision procedure rather than a weighted composite score. Genes identified by both conjFDR and PLACO within the same PS–metabolic trait pair were assigned to the primary priority tier. Within this tier, genes were selected for detailed discussion when at least one peer-reviewed experimental or clinical study linked them to PS and at least one linked them to MetS or the corresponding metabolic component. When multiple genes met these criteria, recurrence across trait pairs and nonredundant representation of metabolic components were used as tie-breakers. This procedure was intended to prioritize representative genes for biological interpretation and was not considered evidence of causality.

### 2.8. Spatial Transcriptomic Enrichment Analysis

To explore the spatial enrichment of trait-associated genetic signals and prioritize potentially relevant cellular populations in PS and MetS, the gsMap (https://github.com/JianYang-Lab/gsMap) approach was applied [20]. gsMap integrates spatial transcriptomics (ST) data with GWAS summary statistics to evaluate whether genetic variants proximal to genes with high expression at defined spatial locations exhibit enrichment for associations with the target trait. The analytical workflow comprises: (1) integration of GWAS data, spatial transcriptomics datasets (including gene expression matrices and spatial coordinates), and LD reference panels; (2) application of graph neural networks to cluster phenotypically homogeneous cells and to compute gene-specific scores, thereby improving signal detection while reducing stochastic noise; and (3) calculation of association significance between each spatial location and the target trait to prioritize potentially relevant cell types and characterize their spatial distribution. By complementing single-cell RNA sequencing with spatial context, gsMap enables exploratory characterization of trait-associated spatial enrichment patterns at single-cell resolution. As the reference atlas was derived from E16.5 mouse embryonic tissues, these patterns were interpreted as hypothesis-generating evidence for tissue and cell-type prioritization, with future validation in disease-matched adult human tissues expected to further clarify their relevance to PS and MetS.

## 3. Results

### 3.1. Genetic correlation

Integration of results from the three genetic correlation analytical methods (LDSC, GNOVA, and HDL) supported significant genome-wide genetic correlations between PS and MetS and several, but not all, of its metabolic components (Table 1). Regarding correlation magnitude, PS demonstrated the strongest positive genetic correlation with MetS overall, with genetic correlation coefficients (rg) estimated as 0.262 (LDSC, *P* = 5.406e-12), 0.2132 (GNOVA, *P* = 9.5356e-26), and 0.3083 (HDL, *P* = 6.12e-13). At the level of individual metabolic components, WC showed the strongest correlation with PS after MetS overall, with rg values of 0.2333 (LDSC, *P* = 1.6046e-22), 0.1911 (GNOVA, *P* = 1.7245e-33), and 0.3015 (HDL, *P* = 3.10e-22). TG also exhibited a consistent positive genetic correlation with PS, with estimates across methods ranging from 0.1223 to 0.1751 (LDSC: rg = 0.1581, *P* = 1.5025e-06; GNOVA: rg = 0.1223, *P* = 4.4945e-08; HDL: rg = 0.175, *P* = 2.23e-06). Notably, HDL-C was the only metabolic component displaying a negative genetic correlation with PS, with rg values ranging from –0.1284 to –0.2286 (LDSC: *P* = 2.054e-08; GNOVA: *P* = 1.6007e-09; HDL: *P* = 1.45e-05). Hypertension demonstrated a weaker yet statistically significant genetic association with PS, with rg values between 0.0573 and 0.137 (LDSC: *P* = 5.433e-08; GNOVA: *P* = 0.0066; HDL: *P* = 3.15e-05). It is noteworthy that the genetic correlation between FBG and PS failed to reach statistical significance in either the LDSC (*P* = 0.0761) or HDL (*P* = 0.5110) analyses (Table 1).

**Table 1:** Genetic correlations and sample-overlap correlations between PS and MetS and its components.

Trait1	Trait2	LDSC-Rg	LDSC-P	intercept (SE)	GNOVA-Rg	GNOVA-P	HDL-Rg	HDL-P	Z-score correlation
MetS	PS	0.262	5.406e-12	−0.0020 (0.0061)	0.2132	9.5356e-26	0.3083	6.12e-13	0.012
FBG	PS	−0.0716	0.0761	0.0125 (0.0053)	0.1015	0.0001	−0.0293	5.11e-01	0.006
HDL-C	PS	−0.1786	5.5823e-09	−0.0044 (0.0069)	−0.1284	1.6007e-09	−0.2286	1.45e-05	0.020
Hypertension	PS	0.0908	0.0067	0.0038 (0.0069)	0.0573	0.0066	0.137	3.15e-05	0.018
TG	PS	0.1593	2.9273e-06	−0.0030 (0.0080)	0.1223	4.4945e-08	0.175	2.23e-06	0.020
WC	PS	0.2352	4.7699e-22	0.0033 (0.0067)	0.1911	1.7245e-33	0.3015	3.10e-22	0.019

The cross-trait LDSC intercepts were generally close to zero, and none remained significant after Bonferroni correction for six comparisons. The Z-score-based assessment similarly indicated limited potential sample overlap between PS and MetS or its five components, with pairwise Z-score correlations ranging from 0.006 to 0.020, all below the prespecified threshold of 0.05. PS: psoriasis; MetS: metabolic syndrome; FBG: fasting blood glucose; HDL-C: high-density lipoprotein cholesterol; TG: triglyceride; WC: waist circumference; LDSC, linkage disequilibrium score regression; GNOVA, genetic covariance analysis; HDL, high-definition likelihood; Z-score correlation: pairwise correlation between GWAS Z-scores used to assess potential sample overlap.

### 3.2. Local genetic correlation

Local genetic correlation analyses were conducted to systematically characterize chromosomal region–specific genetic association patterns between PS and MetS, as well as its five core components, revealing a complex, multilayered genetic interplay.

**Fig 2:**
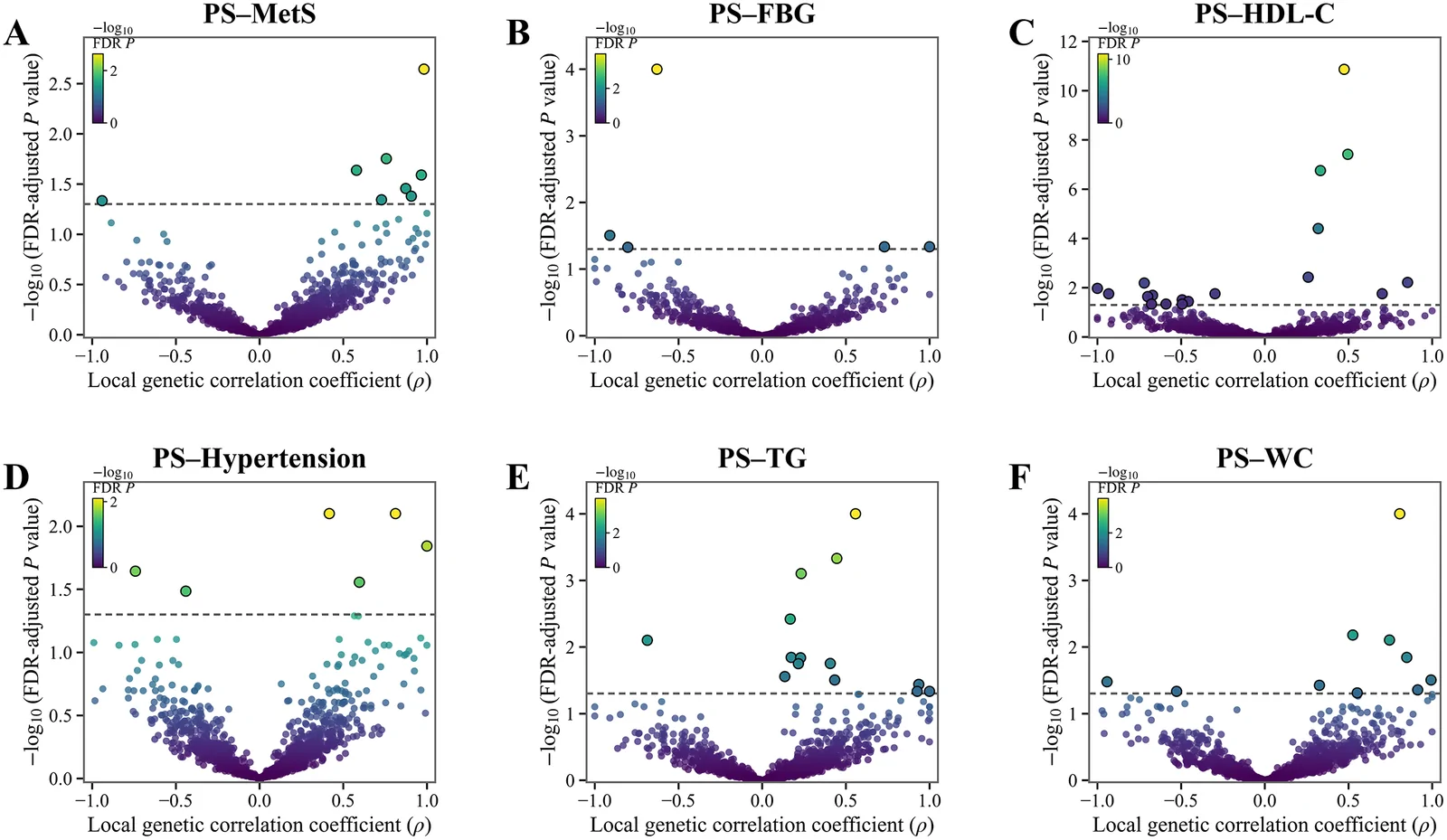
Volcano-style visualization of bivariate local genetic correlation results obtained using LAVA. For each linkage disequilibrium-defined genomic region, the horizontal axis shows the local genetic correlation coefficient (ρ) estimated by the LAVA bivariate analysis, whereas the vertical axis and colour scale show −log_10_-transformed Benjamini–Hochberg false discovery rate (FDR)-adjusted P values. Each point represents one tested local genomic region. The horizontal dashed line denotes the significance threshold of FDR-adjusted P = 0.05 [−log_10_(0.05)], and black-outlined points indicate significant regions. Panels show the local genetic correlations of PS with (A) MetS, (B) FBG, (C) HDL-C, (D) hypertension, (E) TG, and (F) WC. LAVA: local analysis of variant association; FDR: false discovery rate; PS: psoriasis; MetS: metabolic syndrome; FBG: fasting blood glucose; HDL-C: high-density lipoprotein cholesterol; TG: triglyceride; WC: waist circumference.

For PS and overall MetS, eight significant genomic regions were identified, including one negatively correlated region on chromosome 6 and seven positively correlated regions distributed across chromosomes 2, 4, 6, 12, 13, and 20 (Fig 2A; Table 3 in S1 Table). For PS and FBG, five significant segments were detected, comprising two positively correlated segments on chromosomes 3 and 10 and three negatively correlated segments on chromosomes 2, 6, and 15 (Fig 2B; Table 4 in S1 Table). For PS and HDL-C, 18 significant regions were observed, with seven positively correlated regions involving chromosomes 6 and 8 and 11 negatively correlated regions located on chromosomes 3, 5, 6, 8, 10, 12, 16, 18, and 19 (Fig 2C; Table 5 in S1 Table). The hypertension–PS analysis identified six significant segments, including four positively correlated segments on chromosomes 6, 7, 11, and 12 and two negatively correlated segments on chromosomes 11 and 12 (Fig 2D; Table 6 in S1 Table). For TG and PS, 14 significant segments were identified, with one negatively correlated segment on chromosome 17 and the remaining 13 positively correlated segments distributed across chromosomes 2, 6, 11, 12, 13, and 18 (Fig 2E; Table 7 in S1 Table). For WC and PS, 10 significant segments were detected, including two negatively correlated segments on chromosomes 6 and 11 and eight positively correlated segments across chromosomes 2, 6, 10, 11, 12, 16, and 21 (Fig 2F; Table 8 in S1 Table).

Collectively, these findings demonstrate substantial local genetic heterogeneity between PS and MetS and its key components, providing important evidence for shared genetic mechanisms at the regional genomic level.

### 3.3. Polygenic Overlap

**Fig 3:**
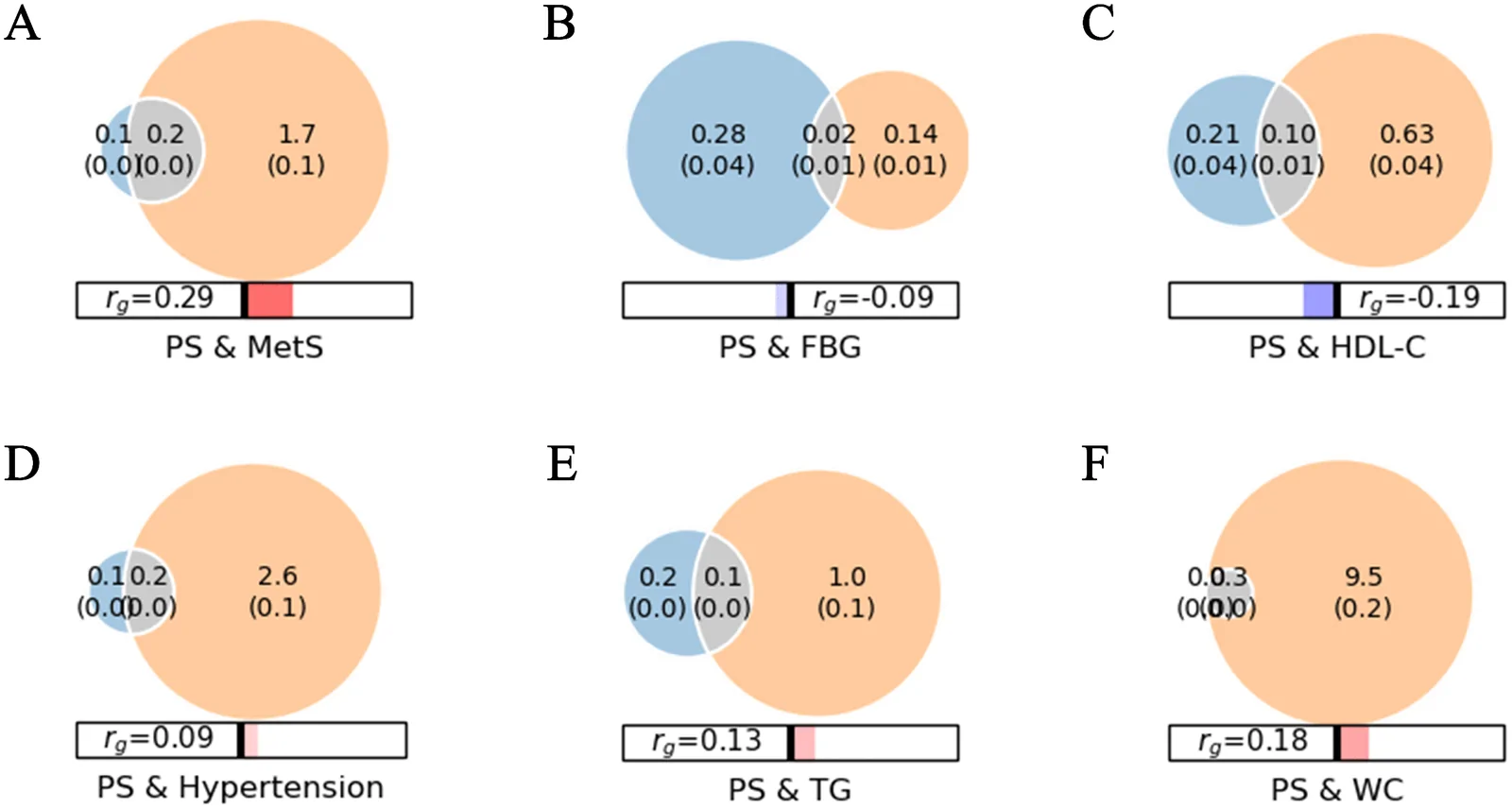
Polygenic overlap between PS and metabolic traits estimated by MiXeR. Venn diagrams display overlapping and distinct “causal” genetic variant sets for trait pairs: (A) PS–MetS, (B) PS–FBG, (C) PS–HDL-C, (D) PS–Hypertension, (E) PS–TG, and (F) PS–WC. Numbers indicate the estimated numbers of variants, in thousands, within each shared or trait-specific component, with standard errors shown in parentheses. Together, these variants explain 90% of the SNP-attributable heritability of each phenotype. Circle proportions reflect the magnitude of the polygenic architecture. The bar beneath each Venn diagram shows the estimated genome-wide genetic correlation (r_g) for the corresponding trait pair. MiXeR: bivariate causal mixture model; SNP: single-nucleotide polymorphism; PS: psoriasis; MetS: metabolic syndrome; FBG: fasting blood glucose; HDL-C: high-density lipoprotein cholesterol; TG: triglyceride; WC: waist circumference.

MiXeR revealed polygenic overlap between PS and MetS and its components. Variant counts represent components explaining 90% of SNP-attributable heritability. For PS–MetS, the Dice coefficient was 0.207 (95% CI, 0.176–0.239), with 0.236k shared variants (SE = 0.023k), r_g = 0.286, and 90.49% concordant effects (Fig 3A; Table 9 in S1 Table). Corresponding estimates were Dice = 0.101 (95% CI, 0.059–0.143), 0.024k shared variants (SE = 0.005k), r_g = −0.088, and 16.66% concordance for PS–FBG (Fig 3B). For PS–HDL-C, Dice was 0.192 (95% CI, 0.150–0.233), with 0.099k shared variants (SE = 0.012k), r_g = −0.191, and 12.80% concordance (Fig 3C). For PS–hypertension, Dice was 0.114 (95% CI, 0.059–0.168), with 0.177k shared variants (SE = 0.045k), r_g = 0.087, and 66.94% concordance (Fig 3D). For PS–TG, Dice was 0.165 (95% CI, 0.106–0.223), with 0.119k shared variants (SE = 0.022k), r_g = 0.133, and 74.14% concordance (Fig 3E). For PS–WC, Dice was 0.061 (95% CI, 0.046–0.076), with 0.307k shared variants (SE = 0.039k), r_g = 0.177, and 99.97% concordance (Fig 3F). However, all four AIC/BIC comparisons were negative and ρβ approached its boundary, indicating limited model support and warranting cautious interpretation. All models summarized 20 subset fits with finite likelihood profiles. Best-versus-minimum AIC supported five pairs except PS–WC, whereas the corresponding BIC values were negative for all pairs. Full-precision shared and trait-specific estimates, Dice confidence intervals, likelihood-profile summaries, and AIC/BIC diagnostics are provided in Table 9 in S1 Table.

**Fig 4:**
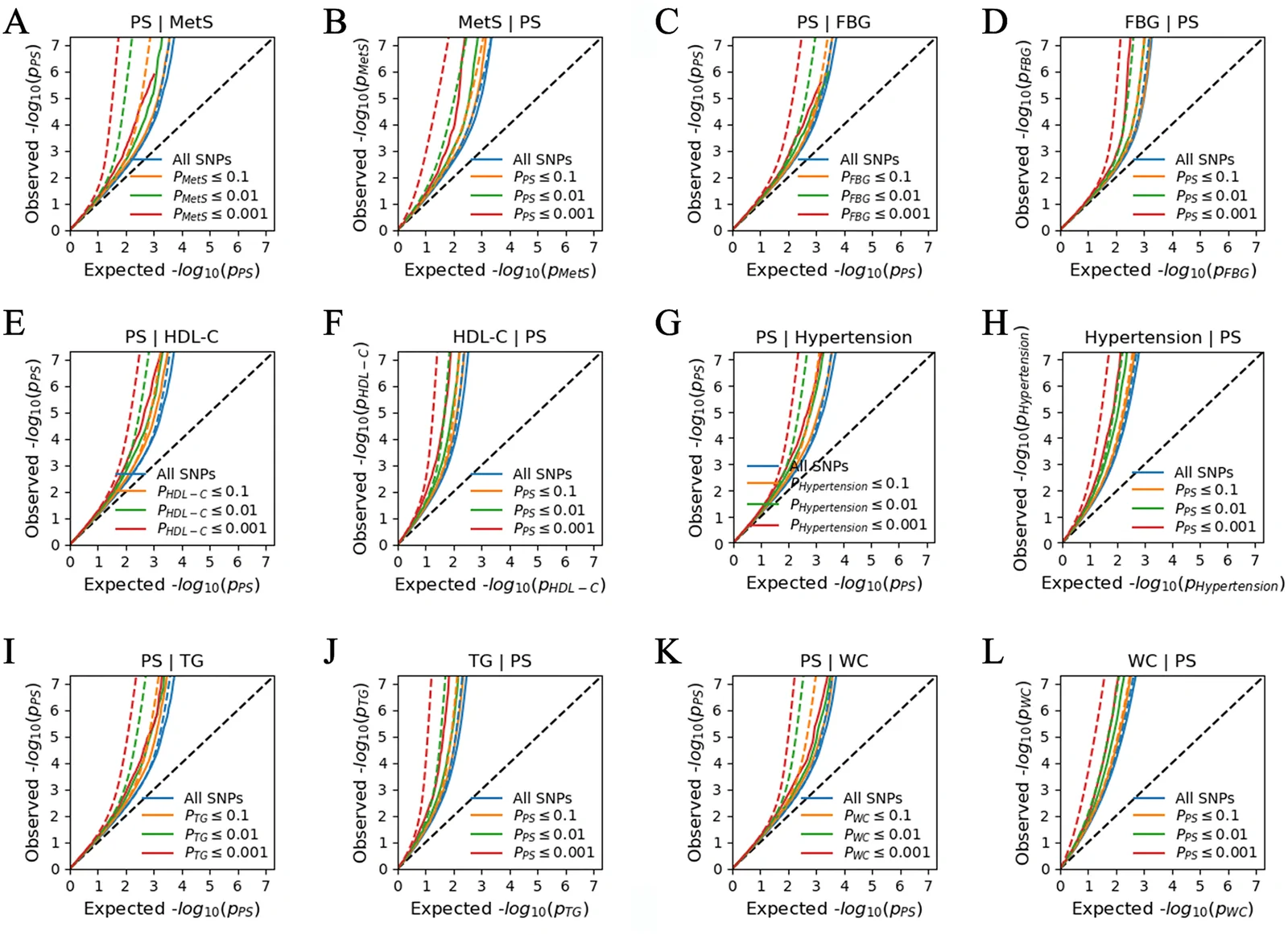
Conditional Q–Q plots showing cross-trait enrichment between PS and MetS and its components. Panels display observed versus expected −log_10_(P) values for the index phenotype conditional on associations with the secondary phenotype. SNPs were grouped using nominal secondary-trait P-value strata: all SNPs (P ≤ 1), P ≤ 0.1, P ≤ 0.01, and P ≤ 0.001. These cutoffs were used for conditional stratification and were not multiple-testing-adjusted significance thresholds. The black diagonal dashed line indicates the expected distribution under the global null hypothesis. Successive leftward deflections with increasingly stringent conditioning strata indicate greater cross-trait enrichment. Panels show reciprocal conditional analyses: (A) PS | MetS, (B) MetS | PS, (C) PS | FBG, (D) FBG | PS, (E) PS | HDL-C, (F) HDL-C | PS, (G) PS | hypertension, (H) hypertension | PS, (I) PS | TG, (J) TG | PS, (K) PS | WC, and (L) WC | PS. The notation X | Y denotes association enrichment for the index phenotype X conditional on associations with phenotype Y. PS: psoriasis; MetS: metabolic syndrome; FBG: fasting blood glucose; HDL-C: high-density lipoprotein cholesterol; TG: triglyceride; WC: waist circumference; SNP: single-nucleotide polymorphism; conjFDR: conjunctional false discovery rate.

Conditional Q–Q plots showed greater cross-trait enrichment under stricter conditioning (Fig 4), supporting shared genetic architecture, although model support was weakest for PS–WC.

### 3.4. Identification of Shared Genomic Loci Between Traits

**Fig 5:**
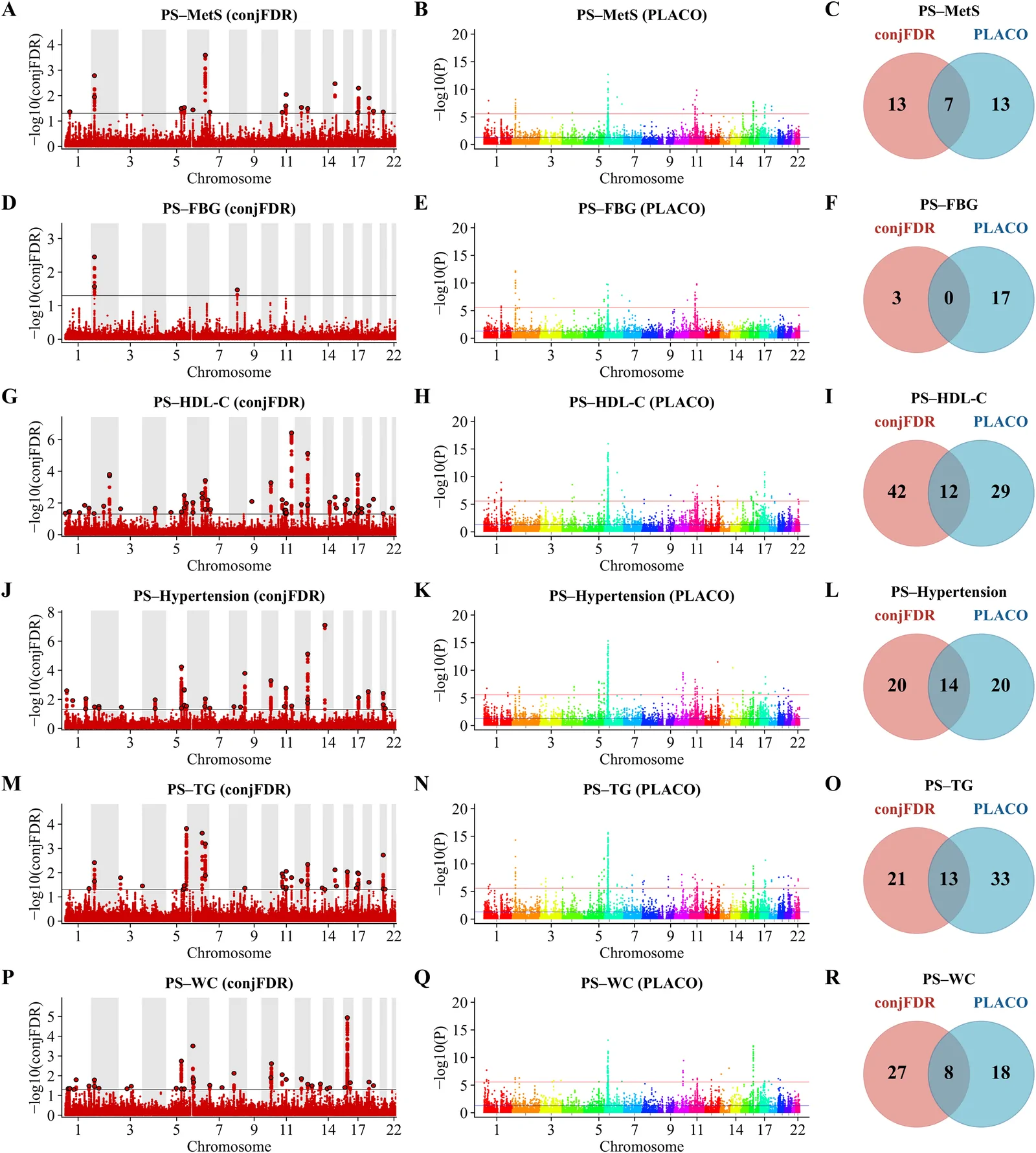
Shared genetic loci and candidate genes identified by conjFDR and PLACO. Panels present conjFDR Manhattan plots, PLACO Manhattan plots, and gene overlaps, respectively, for PS–MetS [(A), (B), (C)], PS–FBG [(D), (E), (F)], PS–HDL-C [(G), (H), (I)], PS–hypertension [(J), (K), (L)], PS–TG [(M), (N), (O)], and PS–WC [(P), (Q), (R)]. Each Manhattan point represents an SNP positioned by chromosome. The conjFDR panels show −log_10_(conjFDR); the grey dashed line marks conjFDR = 0.05 (FDR = 5%), and black outlines denote lead variants. The PLACO panels show −log_10_(P); the red line marks the Bonferroni-corrected genome-wide threshold of P = 5 × 10 ^−^ ⁸ (FWER = 5%), and the blue line marks nominal P = 0.05. Candidate genes were mapped from significant loci using FUMA SNP2GENE. In the Venn diagrams, salmon and blue represent genes identified by conjFDR and PLACO, respectively; grey overlaps indicate genes supported by both methods, and numbers indicate gene counts. condFDR/conjFDR: conditional/conjunctional false discovery rate; PLACO: pleiotropic analysis under composite null hypothesis; FUMA: Functional Mapping and Annotation; SNP: single-nucleotide polymorphism; FDR: false discovery rate; FWER: family-wise error rate; PS: psoriasis; MetS: metabolic syndrome; FBG: fasting blood glucose; HDL-C: high-density lipoprotein cholesterol; TG: triglyceride; WC: waist circumference.

Two analytical strategies, conjFDR and PLACO, were applied to systematically identify the shared genetic basis between PS and MetS and their core components at the genome-wide level. The Z-score-based assessment provided no evidence of significant sample overlap between psoriasis and either metabolic syndrome or its five core components (all P < 0.05; Table 1). For the overall phenotype of PS and MetS, condFDR/conjFDR analysis detected 20 shared susceptibility genes (Fig 5A; Table 10 in S1 Table), and PLACO analysis similarly identified 20 candidate genes (Fig 5B; Table 11 in S1 Table). Seven genes were jointly validated by both methods: *RP11-114H7.1*, *AC008703.1*, *RP11-12A2.3*, *FADS2*, *FOSL1*, *KLF13*, and *RP11-795H16.2* (Fig 5C). For the FBG component, conjFDR and PLACO analyses identified 3 (Fig 5D; Table 11 in S1 Table) and 17 (Fig 5E; Table 13 in S1 Table) candidate genes, respectively, although no cross-validated genes were obtained (Fig 5F). The condFDR/conjFDR analysis for HDL-C detected 57 shared genes (Fig 5G; Table 14 in S1 Table), among which 12 were validated by PLACO analysis: *HEYL*, *IFIH1*, *PPARG*, *HSPA4*, *TRAF3IP2*, *RP11-12A2.3*, *DDX58*, *FOSL1*, *ZC3H12C*, *KLF13*, *EIF3J-AS1*, and *RMI2* (Fig 5H–I; Table 15 in S1 Table). For the hypertension component, both condFDR/conjFDR (Fig 5J; Table 16 in S1 Table) and PLACO analyses (Fig 5K; Table 17 in S1 Table) identified 35 shared genes, with 14 receiving dual validation: *MTHFR*, *PUS10*, *KAT2B*, *MANBA*, *IRF1*, *HSPA4*, *RP11-32D16.1*, *EBF1*, *RGS14*, *RP11-12A2.3*, *RP11-89M16.1*, *CUX2*, *NFKBIA*, and *NFATC2* (Fig 5L). The condFDR/conjFDR (Fig 5M; Table 18 in S1 Table) and PLACO analyses (Fig 5N; Table 19 in S1 Table) for the TG component detected 34 and 46 shared genes, respectively, with 13 cross-validated genes: *RGS12*, *TIMD4*, *LMAN2*, *TRAF3IP2-AS1*, *RP11-12A2.3*, *BDNF*, *ZC3H12C*, *ATXN2*, *KLF13*, *STX1B*, *WNK4*, *RP11-795H16.2*, and *C20orf112* (Fig 5O). For the WC component, condFDR/conjFDR (Fig 5P; Table 20 in S1 Table) and PLACO analyses (Fig 5Q; Table 21 in S1 Table) identified 35 and 26 shared genes, respectively, with 8 validated genes: *RAPGEF6*, *GRM4*, *LRFN2*, *CAMK2G*, *ZMIZ1*, *BDNF*, *FOSL1*, and *FBXL19* (Fig 5R). Collectively, these results indicate extensive and component-specific shared genetic architecture at the gene level between PS and MetS, providing genetic evidence suggestive of potentially shared biological processes.

Application of the hierarchical decision procedure prioritized five genes for focused biological interpretation. *FOSL1* and *FADS2* were prioritized for PS–MetS, *NFKBIA* for PS–hypertension, *BDNF* for PS–TG and PS–WC, and *CAMK2G* for PS–WC. All five genes were supported by both locus-level methods and met the literature-based relevance criterion. *FOSL1* and *BDNF* received additional support through recurrence across multiple trait pairs.

### 3.5. Spatial transcriptomic enrichment patterns

**Fig 6:**
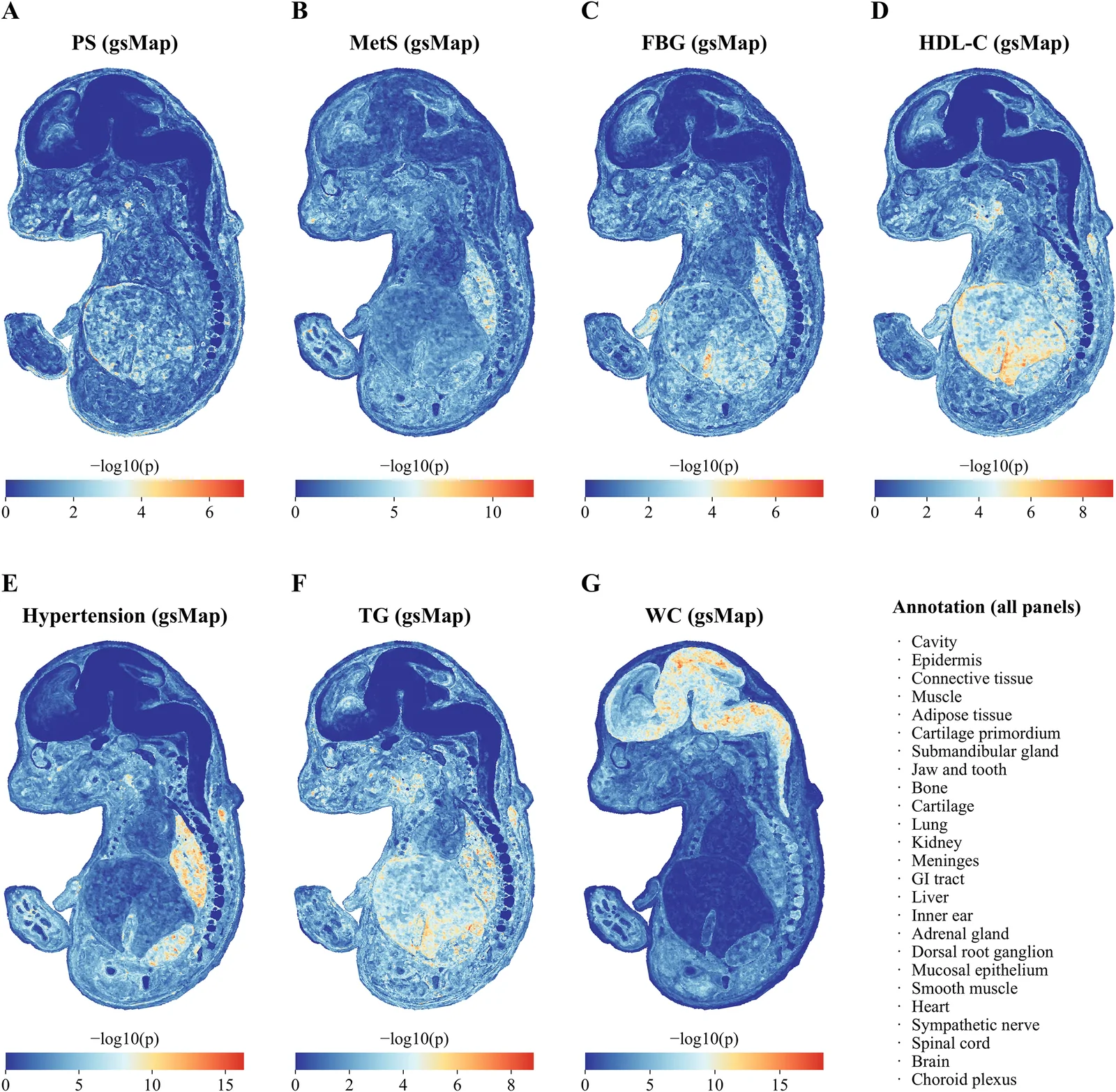
Spatial distribution of trait-associated cellular patterns across 25 organs identified by gsMap. Analyses used single-cell spatial transcriptomics (ST) data from embryonic day 16.5 (E16.5) mouse embryos. Panels depict (A) PS, (B) MetS, (C) FBG, (D) HDL-C, (E) hypertension, (F) TG, and (G) WC. Each spatial spot is coloured by the − log_10_(P) value from a one-sided z-test of S-LDSC enrichment. Blue indicates weaker evidence, whereas yellow-to-red indicates stronger enrichment. P values were corrected across spatial spots, and an FDR-adjusted P value < 0.05 was considered significant. All 25 annotated organs were displayed without preselection, and the anatomical distribution of significant spots was used to prioritize potentially enriched tissues. Colour scales are trait-specific and should be interpreted within each panel. gsMap: genetically informed spatial mapping of cells for complex traits; S-LDSC: stratified linkage disequilibrium score regression; FDR: false discovery rate; PS: psoriasis; MetS: metabolic syndrome; FBG: fasting blood glucose; HDL-C: high-density lipoprotein cholesterol; TG: triglyceride; WC: waist circumference.

The gsMap algorithm was applied to explore the global spatial enrichment of genetic signals associated with PS, MetS, and their core components in an E16.5 mouse embryo atlas. Fig 6 visualizes these trait-associated spatial enrichment patterns at single-cell resolution, providing a developmental spatial framework for prioritizing potentially relevant tissues and cellular populations.

The ST analysis further indicated that trait-associated genetic signals showed pronounced tissue-specific enrichment across multiple embryonic organs. For PS, 14 statistically significant enriched spatial regions were identified, with the top three being the epidermis (pCauchy = 1.0573e-04), adipose tissue (pCauchy = 1.5366e-04), and liver (pCauchy = 1.0167e-03) (Fig 6A; Table 22 in S1 Table). For MetS, FBG, HDL-C, hypertension, and TG, 24, 19, 20, 20, and 20 significantly enriched spatial regions, respectively, were detected, primarily involving the liver, adipose tissue, and epidermis (Fig 6B–F; Tables 23–27 in S1 Table). For WC, 20 significantly enriched spatial regions were identified; enrichment was observed predominantly in adipose tissue (pCauchy = 1.7823e-04), with no significant enrichment detected in the liver or epidermis (Fig 6G; Table 28 in S1 Table). These findings provide hypothesis-generating evidence for prioritizing tissues potentially relevant to PS and MetS, with future validation in disease-matched adult human tissues expected to further clarify their biological relevance.

## 4. Discussion

This study characterized the genetic associations between PS, MetS, and its core components using complementary approaches, including LDSC, GNOVA, HDL, LAVA, MiXeR, conjFDR, PLACO, and gsMap. Significant genome-wide genetic correlations were identified, while local analyses mapped shared genetic signals to specific chromosomal regions. Integrative analyses further prioritized candidate genes and identified spatial enrichment patterns of disease-associated cells. These findings provide insight into genetic factors associated with the coexistence of PS and MetS. However, MetS is a composite clinical construct, and differences in diagnostic criteria across source GWASs may contribute to phenotype heterogeneity. Moreover, WC, TG, HDL-C, FBG, and hypertension are related but distinct cardiometabolic endophenotypes with partially different genetic architectures. Thus, loci shared between PS and individual components may reflect broader cardiometabolic susceptibility rather than MetS-specific biology. Interpretation should therefore consider both component-specific and cross-component patterns, with functional validation required to establish biological specificity.

Current clinical epidemiological evidence has consistently demonstrated significant positive associations between PS and MetS as well as its constituent components. A large-scale population-based study from Spain, which included 398,701 individuals, reported a 13.2% higher incidence of MetS in PS patients compared with healthy controls [*p* < 0.001, odds ratio (OR): 2.21] [26]. A meta-analysis incorporating 63 studies and pooling data from 15,939 PS patients and 103,984 control subjects further indicated that the prevalence of MetS reached 30.29% in the PS group versus 21.70% in the control group, with the between-group difference being statistically significant [OR: 2.077, 95% confidence interval (CI): 1.84–2.34] [27]. The risk of MetS also demonstrated a graded increase across PS severity levels. In a cohort of 44,715 participants, MetS risks of 22%, 56%, and 98% were observed in patients with mild, moderate, and severe PS, respectively, reflecting a dose-dependent pattern [28]. Regarding glucose metabolism disorders, a prospective cohort study tracking 8,124 PS patients found a 46% elevated risk of developing abnormal FBG [hazard ratio (HR): 1.46, 95% CI: 1.28–1.67], and the cumulative incidence of diabetes increased in parallel with disease duration [29]. The association with lipid metabolism abnormalities has been similarly well established. A cross-sectional survey of 33,588 participants reported that serum TG levels in PS patients were elevated by an average of 23.7 mg/dL relative to controls, with the risk of hypertriglyceridemia increased to 1.72-fold (OR: 1.72, 95% CI: 1.53–1.94). Meanwhile, HDL-C levels showed a marked decreasing trend (mean reduction of 4.2 mg/dL), accompanied by a 1.43-fold higher risk of low HDL-C (OR: 1.43, 95% CI: 1.29–1.59) [30]. With respect to blood pressure indices, a 10-year prospective study of 256,356 adults (42,726 hypertensive patients and 213,630 controls) found that individuals with hypertension had a 54% increased risk of developing PS compared with normotensive subjects (HR: 1.54, 95% CI: 1.47–1.61, *p* < 0.001) [31]. For abdominal obesity, a 2021 cross-sectional study involving 12,502 PS patients documented a central obesity prevalence of 61.3%, substantially exceeding the 38.7% observed in controls (OR: 2.51, 95% CI: 2.31–2.73). Moreover, each 5 cm increase in WC was associated with an average rise of 1.8 points in the Psoriasis Area and Severity Index score [32]. Finally, a recent genetic study employing Mendelian randomization provided causal-level evidence supporting these associations, confirming that MetS, WC, and hypertension each represent independent genetic risk factors for PS development [7]. More recent genetic studies have further refined the epidemiological evidence linking PS with metabolic dysfunction. A 2026 two-sample Mendelian randomization analysis reported that genetically predicted TG, but not total fatty-acid levels, was associated with PS risk [33]. An integrative study combining Mendelian randomization, transcriptomic analysis, and single-cell RNA sequencing subsequently identified shared obesity–PS genes. Functional experiments further supported COX7C as a regulator of keratinocyte proliferation and inflammatory responses [34]. A systematic synthesis of 346 psoriasis-related Mendelian randomization studies also linked disrupted lipid metabolism, particularly elevated LDL-C and selected phospholipid species, to increased PS risk [35]. Together, these findings support metabolically mediated genetic contributions to PS while highlighting exposure-specific and method-dependent heterogeneity. They therefore complement, rather than establish, a single causal explanation for the association between PS and MetS.

The PS–FBG findings require separate and cautious interpretation. Although epidemiological studies have associated psoriasis with dysglycaemia, the genome-wide genetic evidence in this study was inconsistent. LDSC and HDL yielded non-significant negative estimates, whereas GNOVA detected a modest positive correlation. MiXeR likewise estimated a negative genetic correlation and showed that only 16.66% of shared variants had concordant effect directions, indicating predominantly discordant effects. These discrepancies may reflect differences among the methods in their statistical assumptions, LD modelling, effect-size weighting, and sensitivity to phenotype heterogeneity or statistical power. Moreover, the significant local correlations identified by LAVA indicate regional heterogeneity but do not establish a consistent genome-wide relationship. The PS–FBG findings should therefore be considered tentative and component-specific rather than evidence of a robust shared genetic architecture, and they require replication in harmonized independent cohorts.

Among the numerous genes identified, *FOSL1, FADS2, NFKBIA, BDNF*, and *CAMK2G* were prioritized for focused discussion based on a combination of convergent statistical evidence, functional relevance, and previous experimental or clinical findings. Specifically, *FOSL1* and *FADS2* were jointly identified by conjFDR and PLACO for PS–MetS, *NFKBIA* for PS–hypertension, *BDNF* for PS–TG and PS–WC, and *CAMK2G* for PS–WC. These genes were therefore selected as representative, literature-supported examples of the broader shared-locus findings. Among them, *FOSL1* may function as an “inflammatory hub” exerting bidirectional regulatory effects, contributing to both aberrant keratinocyte proliferation and inflammatory responses in PS, while also mediating vascular endothelial dysfunction associated with MetS. *FOSL1* expression is markedly elevated in psoriatic lesions, where it transcriptionally activates *TRAF3* and subsequently initiates the nuclear factor-κB (NF-κB) signaling pathway, thereby modulating excessive keratinocyte proliferation and *NLRP3* inflammasome–driven inflammatory responses [36]. This amplification of inflammatory signaling not only aggravates local immune dysregulation but may also facilitate core pathological processes of MetS, including insulin resistance, vascular endothelial injury, and lipid metabolic disturbances, through the systemic release of inflammatory mediators [37]. *FADS2*, the rate-limiting enzyme of polyunsaturated fatty acid (PUFA) biosynthesis, is significantly downregulated in the keratinocytes of PS patients. Its deficiency disrupts docosahexaenoic acid (DHA) production, enhances inflammatory responses, and contributes to neutrophil recruitment [38]. Reduced FADS2 activity diminishes the synthesis of anti-inflammatory omega-3 long-chain PUFAs (e.g., eicosapentaenoic acid and DHA) while impairing the desaturation of omega-3 fatty acids to DHA, thereby promoting inflammatory signaling through activation of the NF-κB pathway [5]. *NFKBIA* dysfunction results in aberrant activation of NF-κB transcription factors, increasing the transcription of pro-inflammatory cytokines and immune-related genes. This sustained immune activation exacerbates cutaneous inflammation in PS and affects vascular function through systemically circulating inflammatory mediators [39]. *NFKBIA* polymorphisms have been reported to be independently associated with elevated coronary artery disease risk and cardiovascular risk profiles [40], with NF-κB serving as a central inflammatory and immunoregulatory mediator in cardiovascular disease, atherosclerosis, and diabetes [41]. Plasma BDNF levels are markedly reduced in PS patients, accompanied by increased body mass index (BMI), systolic blood pressure, HDL-C, LDL-C, and TG levels [42]. PS patients frequently exhibit hypothalamic–pituitary–adrenal (HPA) axis dysregulation, with bedtime salivary cortisol levels positively correlating with disease severity, whereas stress-induced cortisol elevations are associated with reduced *BDNF* concentrations [43,44]. Experimental studies have demonstrated that *BDNF* administration into the paraventricular nucleus of the hypothalamus significantly reduces serum TG levels in high-fat diet–induced obese rats [45]. *CAMK2* activity is notably elevated in obese adipose tissue, whereas adipocyte-specific *CAMK2* deficiency ameliorates obesity-related glucose intolerance and insulin resistance [46]. *CAMK2G* hyperactivation induces chronic low-grade adipose tissue inflammation, promoting adipocyte dysfunction and lipid accumulation, while simultaneously amplifying psoriatic cutaneous inflammation through systemic release of pro-inflammatory cytokines [47,48]. The additional loci identified in our analyses remain biologically relevant candidates and warrant further functional investigation.

The spatial transcriptomic analysis identified enrichment of trait-associated genetic signals in embryonic epidermal, adipose, and liver regions. These patterns are consistent with potentially related involvement of these tissues in PS and MetS but do not directly demonstrate an inter-organ functional axis. Previous studies have described inflammatory links among the skin, adipose tissue, and liver [49–52]. However, WC-associated enrichment was concentrated predominantly in adipose tissue, indicating that the spatial pattern was not uniform across traits. The gsMap findings should therefore be viewed as supportive of a putative epidermis–adipose–liver relationship and as hypothesis-generating evidence for tissue prioritization, rather than direct evidence of causal inter-organ communication. Validation in disease-matched adult human spatial and functional studies is required.

The novelty of this study lies in integrating genome-wide, local, polygenic, pleiotropic, and spatial analyses within a unified framework. Joint evaluation of MetS and its five components revealed both shared genetic patterns and component-specific heterogeneity. Cross-method prioritization highlighted FOSL1, FADS2, NFKBIA, BDNF, and CAMK2G, while gsMap prioritized embryonic epidermal, adipose, and hepatic regions. Together, these findings provide a multi-level framework for the functional investigation of PS–MetS comorbidity. Despite these contributions, several limitations should be acknowledged. First, heterogeneity in phenotype definitions, genotyping, imputation, cohort selection, and reporting across public datasets may affect genetic correlation and shared-locus estimates. The E16.5 mouse atlas may also not adequately represent adult human disease tissues, making the spatial findings hypothesis-generating. Second, residual long-range LD outside the excluded MHC region may complicate the interpretation of chromosome 6 loci. Third, exact sample overlap was unavailable. Although LDSC intercepts, Z-score correlations, GNOVA, and PLACO suggested limited bias, residual overlap cannot be excluded. Fourth, MiXeR model support was limited: BIC comparisons were negative for all trait pairs, AIC did not support PS–WC, and ρβ approached its boundary. These estimates should therefore be considered descriptive. Fifth, PS–FBG results were inconsistent across methods. GNOVA indicated a modest positive correlation, whereas LDSC and HDL yielded nonsignificant negative estimates and MiXeR suggested predominantly discordant effects. These findings remain tentative despite significant local LAVA signals. Sixth, restriction to European-ancestry cohorts limits generalizability. Seventh, the identified variants lack functional validation. Future studies should replicate these findings in diverse cohorts and validate the spatial signals and prioritized genes in disease-matched human tissues.

## 5. Conclusion

Genetic analyses identified shared architecture between psoriasis and metabolic syndrome, with heterogeneous evidence across individual components. Integrative analyses prioritized shared loci and identified trait-associated spatial enrichment patterns. These findings provide a hypothesis-generating framework for future biological investigation but do not establish causal mechanisms or clinical utility. Development and external validation of polygenic risk scores, followed by prospective clinical evaluation, would be required before considering applications to screening, risk stratification, or individualized intervention.

## Supporting information

S1 Table Comprehensive summary of supplementary results. This Excel workbook contains the complete results of the supplementary analyses in 28 worksheets (Tables 1–28), each corresponding to the relevant in-text citation.
(XLSX)
